# Eyespots deflect predator attack increasing fitness and promoting the evolution of phenotypic plasticity

**DOI:** 10.1098/rspb.2014.1531

**Published:** 2015-01-07

**Authors:** Kathleen L. Prudic, Andrew M. Stoehr, Bethany R. Wasik, Antónia Monteiro

**Affiliations:** 1 Department of Ecology and Evolutionary Biology, Yale University, New Haven, CT, USA; 2 Department of Integrative Biology, Oregon State University, Corvallis, OR, USA; 3 Department of Biology, Butler University, Indianapolis, IN, USA; 4 Department of Ecology and Evolutionary Biology, Cornell University, Ithaca, NY, USA; 5 Department of Biological Sciences, National University of Singapore, Singapore; 6 NUS-Yale College, Singapore

**Keywords:** adaptive coloration, visual signalling, wing patterns

## Abstract

Some eyespots are thought to deflect attack away from the vulnerable body, yet there is limited empirical evidence for this function and its adaptive advantage. Here, we demonstrate the conspicuous ventral hindwing eyespots found on *Bicyclus anynana* butterflies protect against invertebrate predators, specifically praying mantids. Wet season (WS) butterflies with larger, brighter eyespots were easier for mantids to detect, but more difficult to capture compared to dry season (DS) butterflies with small, dull eyespots. Mantids attacked the wing eyespots of WS butterflies more frequently resulting in greater butterfly survival and reproductive success. With a reciprocal eyespot transplant, we demonstrated the fitness benefits of eyespots were independent of butterfly behaviour. Regardless of whether the butterfly was WS or DS, large marginal eyespots pasted on the hindwings increased butterfly survival and successful oviposition during predation encounters. In previous studies, DS *B. anynana* experienced delayed detection by vertebrate predators, but both forms suffered low survival once detected. Our results suggest predator abundance, identity and phenology may all be important selective forces for *B. anynana*. Thus, reciprocal selection between invertebrate and vertebrate predators across seasons may contribute to the evolution of the *B. anynana* polyphenism.

## 1. Introduction

Many organisms have evolved protective coloration to diminish and dissuade predator attack. Eyespot patterns, concentric rings of contrasting colours, are one form of protective coloration against predators. Broadly speaking, large singular eyespots function by startling or intimidating predators while smaller, numerous eyespots at the body margin divert predator attack to less vulnerable body parts (reviewed in [1,2]). Several studies have supported the intimidation hypothesis for eyespots (e.g. [3–6]), yet there is little empirical evidence for the deflection hypothesis (e.g. [7–11]).

Documenting the deflective function of eyespots, specifically ones on the margins of the animal, has proved difficult. Numerous studies suggest eyespots do not re-direct predator attack to the body margin or increase prey survival (e.g. [3–8]). There are a few experiments indirectly suggesting eyespots may be deflective [9,11]. For example, Olofsson *et al*. [9] demonstrated, under very specific lighting conditions of high ultraviolet (UV) and low visible light intensity, birds attack marginal wing eyespots on dead butterflies. The results of these few experiments beg the questions: *can* eyespots deflect predator attack of live prey? and *do*es this deflection impact prey survival and reproduction?

**Figure 1.**
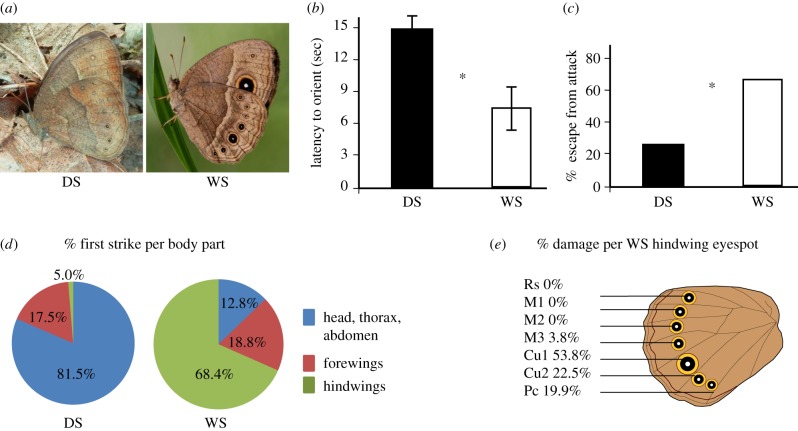
Mantid attack behaviours on dry season (DS) and wet season (WS) forms of *Bicyclus anynana*, and survival outcome for arena experiments. (*a*) The ventral surface of the two seasonal forms of *B. anynana*. Note the differences in the ventral hindwing eyespot size. (*b*) Latency for the invertebrate predator, *Tenodera sinensis*, to orient on each form of *B. anynana*. Means±95% CI presented. The DS form took longer for mantids to detect. (*c*) Percentage of butterfly escape once attacked by a praying mantid. The DS form was much less likely to escape once an attack was initiated. (*d*) Percentage of mantid first strike on various body parts of *B. anynana*. The WS form was attacked more frequently on the hindwings than the DS form. (*e*) Percentage of damage observed per hindwing eyespot in the WS form only. Eyespots Cu1, Cu2 and Pc were the most damaged. (Online version in colour.)

Here, we investigated the deflection hypothesis and its impacts on prey survival and reproduction by assessing the behavioural response of an invertebrate predator to a butterfly species which exhibits a seasonal polyphenism in eyespot size (figure 1*a*). We presented live butterflies (*Bicyclus anynana*) to hand-reared Chinese mantids (*Tenodera sinensis*) in a series of laboratory experiments. In the first experiment, we observed the response of mantids to butterflies with small eyespots and large eyespots, noting the position of their attacks on the butterfly wing and the percentage of prey escapes. Second, we conducted a microcosm experiment with a single predator and multiple butterflies of one seasonal form evaluating both the length of butterfly survival and number of eggs laid. Finally, we controlled for the effect of butterfly behaviour across seasonal forms by transplanting eyespots from one form to the other and repeating the microcosm experiment.

The seasonal eyespot polyphenism of *B. anynana* is determined by early developmental temperature conditions [12]. If the immature experiences ambient temperatures above 23°C, then the adult has large, bright and conspicuous marginal eyespots on its ventral hindwing. If the immature experiences ambient temperatures below 19°C, then the adult has small, dull and cryptic marginal eyespots on its ventral hindwing. This polyphenism is referred to as wet season (WS) and dry season (DS) forms, respectively (figure 1*a*). Previous research demonstrated the smaller, duller DS eyespots are more advantageous against vertebrate predators relative to WS eyespots, by delaying prey detection and increasing latency to attack [8]. However, once the prey is discovered, both DS and WS have a low probability of escape and survival [7,8,10], calling into question the functional benefits and adaptive importance of the WS form.

*Tenodera sinensis*, the Chinese mantid, has a widespread distribution, is neurologically and behaviourally similar to other mantid species, and has been used as a model for investigating insect predator behaviour (e.g. [13,14]). *Bicyclus anynana* inhabits geographical areas with some of the highest global mantid diversity and abundance [15]. Mantids and other invertebrate predators have been observed as active predators in the field with *B. anynana*, especially during the wet season [16] making mantids good candidates as selective agents for butterflies with large eyespots. Here we investigated whether the large marginal eyespots of *B. anynana* WS form deflect mantid attack to the wing margin thereby increasing butterfly survival and reproduction when compared with the reduced margin eyespots of the WS form.

## 2. Material and methods

### (a) Experimental animals

Eggs of *B. anynana* were collected from females from a laboratory colony. Larvae were raised on young maize plants (*Zea mays*) in a climate room at either 27°C (to produce the WS form) or 17°C (to produce the DS form) with a 12 L : 12 D cycle and 80% relative humidity. Males and females were separated on the day of pupal eclosion so they were virgins at the onset of the experiments. All adults were kept at 22°C and fed a maintenance diet of banana.

Egg cases of *T. sinensis* were purchased from Carolina Biological Supply Company and reared to adults in individual cages on a successive diet of fruit flies, houseflies and crickets. They were raised to the ultimate instar at 27°C (wet season temperatures) with a 12 L : 12 D cycle and 80% relative humidity. Mantids were not exposed to either butterflies or eyespots before any trial.

### (b) Mantid response to individual wet season and dry season prey in an arena

The arena was designed to ritualize the encounter between prey and predator across trials; it consisted of three components: a rectangular ramp, a square floor and a cylindrical wall (electronic supplementary material, figure S1*a*). The ramp began outside the cylindrical wall, continued through a port in the wall, and continued upwards inside the wall at an 18° angle measuring a total of 35 cm long and 7 cm wide. Both ramp and floor were constructed of wood and covered in poster board paper, while the cylindrical wall was constructed of paper. Ramp, floor and wall were painted a uniform green (paint reflectance spectra in the electronic supplementary material, figure S1*b*). The arena was illuminated by two full-spectrum halogen lamps (Solux-Eiko 18003, 50W, 4700°K, CRI 91, 36° field of illumination; see irradiance spectra in the electronic supplementary material, figure S1*c*). Each lamp was positioned 23 cm above the highest point of the ramp and 20 cm from the other lamp to fully illuminate the arena.

A single butterfly was starved for 24 h then placed at the top of the ramp inside the arena wall. The posterior/anterior axis of the butterfly was perpendicular to the ramp. Butterflies were trained to remain in this position by a small food reward, a small piece of banana placed on a piece of removable parafilm. At the start of each trial, the butterfly acclimated for 5 min before the predator was introduced. After 5 min, a single mantid was placed at the ramp base outside the arena wall, out of view of the butterfly. Mantids are negatively geotatic, walking upwards from a lower position and were previously trained via a cricket food reward to walk directly up the ramp at a steady pace without stopping. Once a mantid demonstrated the desired walking behaviour consistently three times in a row, experimental trials with butterflies began. All mantids learned to walk up the ramp, without straying or stopping, over the course of three to five experiences until they completed the three trial series.

We used a repeated-measures design where each mantid (*n* = 20) encountered both WS and DS butterflies in random order. Each mantid experienced each butterfly form four times, a new butterfly was used for each trial. We measured latency to orient on prey, latency to attack prey and prey survival after attack. Almost all trials resulted in a mantid attack sequence, but in two trials the butterfly flew away before an attack was initiated (*n* = 2 out of 160 trials, 1% of trials resulted in no attack). These two butterflies were then removed from the experiment and the mantids were given another trial with a different butterfly to have even numbers of trials across mantids. Data were either square root (days, eggs) or arcsine transformed (per cent wing damage), evaluated for homoscedasticity and sphericity then analysed using one-way repeated measures ANOVA (R v. 2.13.0) with mantid as a random effect. We present *p*-values from two-tailed tests with *α* = 0.05. Means and 95% CI are reported.

We also noted the attack location on the butterfly. Because mantids remove the wings of butterflies before consuming them, we were able to record eyespot damage for both killed and escaped butterflies. WS wing damage data were evaluated with *t*-tests. We present *p*-values from two-tailed tests with *α* = 0.05.

### (c) Butterfly polyphenism and predation microcosm experiment

We used a 2 × 2 factorial design (mantid presence × butterfly form) to evaluate the effect of seasonal form on butterfly survival and reproduction in the presence of a mantid predator. There were four treatments: without mantid × DS butterflies, with mantid × DS butterflies, without mantid × WS butterflies and with mantid × WS butterflies. There were 12 trials per treatment (*n* = 48), each conducted under full-spectrum lights at 22°C. Each trial was performed in a microcosm cylindrical cage (50 cm diameter × 100 cm height) with a full-spectrum light source, adult food source and oviposition substrate (*Zea mays*). We used 10 virgin butterflies (five males and five females) and 0–1 mantid in each trial. All experimental animals were naive, i.e. did not experience each other before the trial. Trials began at 12.00 and lasted until all the butterflies were consumed. We counted the number of half days until all the *B. anynana* were consumed by the mantid. We also recorded the amount of wing damage on the remaining wings left by the mantids and the number of eggs laid by the five females in each cage. Data were transformed, evaluated for homoscedasticity and sphericity then analysed using linear models with an interaction (R v. 2.13.0). We present *p*-values from two-tailed tests with *α* = 0.05. Means and 95% CI are reported.

### (d) Eyespot manipulation and predation microcosm experiment

The developmental polyphenism of *B. anynana* alters multiple traits other than eyespot size and brightness which may influence predator–prey dynamics [17,18]. WS butterflies may be able to anticipate and escape a predator attack better than DS butterflies, independently of their wing pattern because they are more active than DS at the same ambient temperature and light environment [19]. Additionally, WS and DS butterflies have different eye sizes and ommatidia dimensions, suggesting visual plasticity between forms [20]. We used a 2 × 2 factorial design (butterfly form × eyespot form) to evaluate the effect of ventral wing eyespots on butterfly survival and reproduction in the presence of a mantid predator. We transplanted a small strip of the ventral hindwing margin with all the eyespots to each butterfly securing the wing piece with Superglue. The transverse white band of the two seasonal forms was not transferred. There were four treatments, all with mantids: DS butterfly × DS eyespots, DS butterfly × WS eyespots, WS butterfly × DS eyespots and WS butterfly × WS eyespots. All other experimental and analyses protocols were identical to the polyphenism and predation microcosm experiment above.

## 3. Results and discussion

### (a) Eyespots on wet season butterflies increase prey detectability, influence attack location and increase survival

In the arena experiment, we found WS butterflies were easier for predators to detect, but harder to capture and more likely to escape with eyespot damage (figure 1). Mantids oriented on WS butterflies much sooner (DS 16.9 ± 4.3 s, WS 8.5 ± 3.1 s, *F*_1,19_ = 9.18, *p* = 0.0016; figure 1*b*). Many more WS butterflies escaped once attacked by mantids (DS 26.1% escaped, WS 69.2% escaped *F*_1,19_ = 6.59, *p* = 0.0015; figure 1*c*; electronic supplementary material, video). We observed 68.8% of attacks on the margin of the ventral hindwing of WS butterflies compared with 5.0% on the wing margin of DS butterflies (*F*_1,19_ = 8.71, *p* < 0.001; figure 1*d*; electronic supplementary material, video). Within WS, the ventral hindwing Cu1 eyespot received the most damage (53.8%) followed by Cu2 (22.5%) and Pc (19.9%) (*F*_1,9_ = 7.52, *p* < 0.001; figure 1*e*).

### (b) Wet season butterflies live longer and reproduce more with mantids

**Figure 2.**
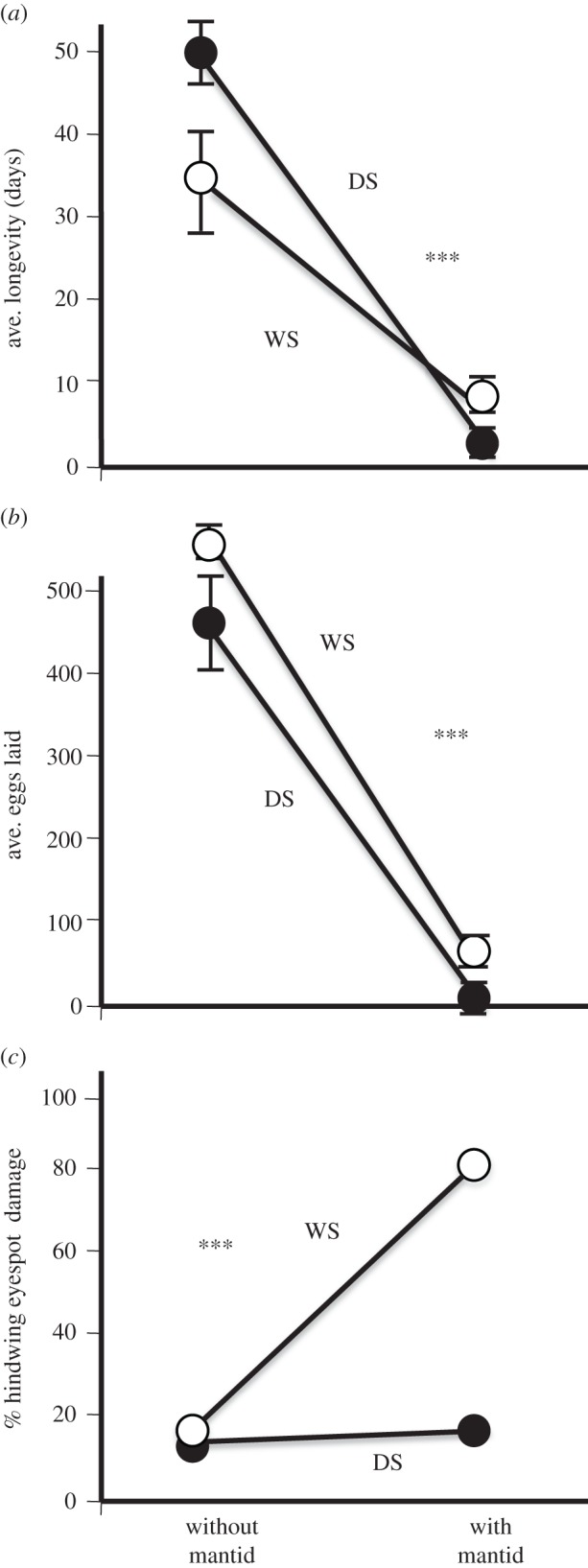
Longevity, fecundity and incurred wing damage for the DS and WS forms of *B. anynana* in microcosm experiments in the presence or the absence of mantids. (*a*) Average longevity measured in days. Means ± 95% CI are reported. DS forms survived longer in the absence of mantids while WS forms survived longer in the presence of mantids. (*b*) Average number of eggs laid. Means ± 95% CI are reported. WS form females laid more eggs, and mantids negatively impacted oviposition in both forms. (*c*) Percentage hindwing eyespot damage. WS forms experienced greater amounts of damage on their ventral hindwing eyespots in the presence of a mantid. Asterisks indicate statistical significance between the treatments.

WS butterflies survived longer, laid more eggs and suffered more hindwing damage when mantids were present (figure 2). Both butterfly forms survived longer without mantids (without mantid 42.9 ± 2.6; with mantid 4.8 ± 1.9 days, *F*_1,44_ = 442.08, *p* < 0.0001), and both forms lived a similar number of days controlling for predator treatments (*F*_1,44_ = 0.09, *p* = 0.7595). Mantids negatively impacted DS butterflies more; DS had lower survival than WS (DS with mantid 2.3 ± 1.0 days, WS with mantid 7.4 ± 2.8 days, *F*_1,44_ = 63.76, *p* < 0.0001; figure 2*a*). Both butterfly forms laid more eggs without mantids (without mantid 510.3 ± 13.6 eggs, with mantid 18.5 ± 2.4 eggs, *F*_1,44_ = 1230.75, *p* < 0.0001). Across predator treatments, WS laid more eggs than DS (DS 469.2 ± 15.3 eggs, WS 550.9 ± 19.4 eggs, *F*_1,44_ = 14.35, *p* = 0.001) as noted in previous studies [17,18]. Within mantids, WS butterflies laid more eggs (DS 6.7 ± 2.3 eggs, WS 31.0 ± 3.4 eggs, *F*_1,22_ = 14.35, *p* = 0.001). There was no interaction effect between predator presence and butterfly form (*F*_1,44_ = 2.64, *p* = 0.1060; figure 2*b*). Both butterfly forms suffered less ventral hindwing damage without mantids (without mantid 8.9 + 0.4%, with mantid 44.6 ± 0.3%, *F*_1,44_ = 702.96, *p* < 0.0001). Across predator treatments, WS butterflies suffered more ventral hindwing damage than DS (DS 13.4 ± 3.9%, WS 59.5 ± 3.7%, *F*_1,44_ = 467.14, *p* < 0.001). Mantids damaged WS butterflies more (DS 10.6 ± 1.9%, WS 70.4 ± 2.1%, *F*_1,44_ = 350.92, *p* < 0.0001; figure 2*c*).

### (c) Eyespots alone deflect attack and increase butterfly fitness

**Figure 3.**
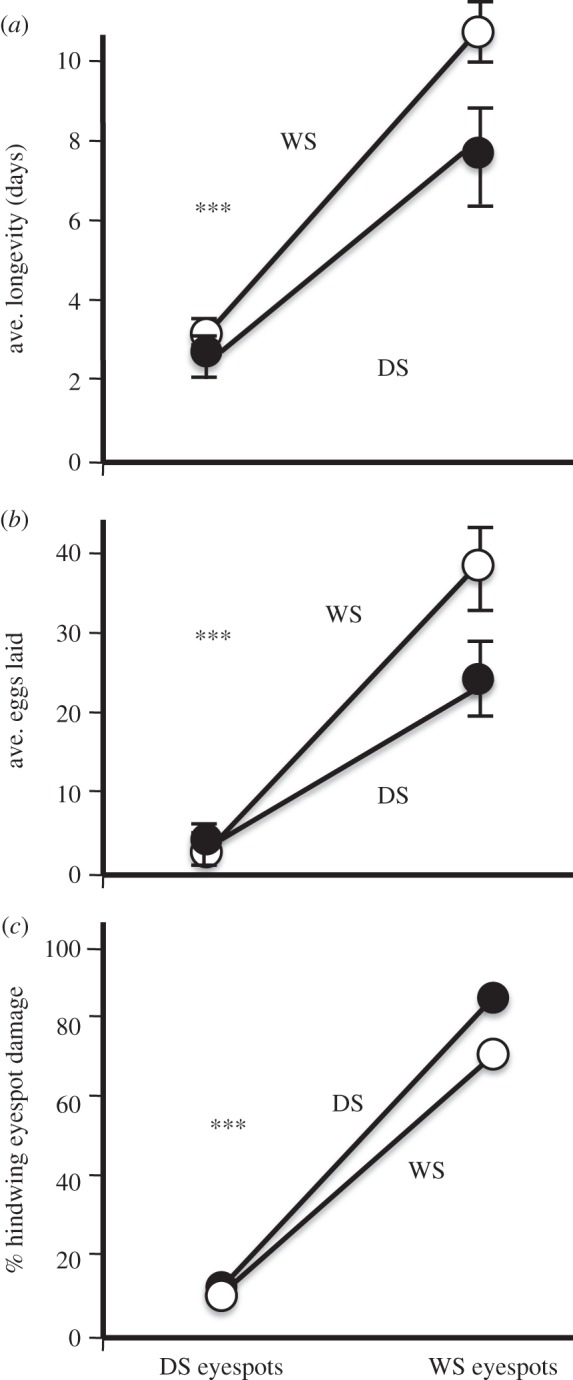
Longevity, fecundity and incurred wing damage for the DS and WS forms of *B. anynana* with transplanted ventral hindwing eyespots in microcosm experiments with mantids. (*a*) Average longevity measured in days. Means ± 95% CI are reported. Butterflies with WS eyespots survived much longer regardless of butterfly form. (*b*) Average number of eggs laid. Means ± 95% CI are reported. Butterflies with WS eyespots laid more eggs regardless of butterfly form. (*c*) Percentage hindwing eyespot damage. Butterflies with WS eyespots, regardless of butterfly form, exhibited greater amounts of damage on their ventral hindwing eyespots. Asterisks indicate statistical significance between the treatments.

WS eyespots, regardless of the form they were attached to, increased butterfly survival, reproductive output and ventral hindwing damage (figure 3). WS butterflies, controlling for eyespot treatment, survive longer than DS (DS form 5.1 ± 0.6 days, WS form 7.0 ± 0.3 days, *F*_1,44_ = 9.59, *p* = 0.0034). WS eyespots, regardless of the form they were attached to, increased butterfly longevity (DS eyespots 2.5 ± 0.4 days, WS eyespots 9.6 ± 0.3 days, *F*_1,44_ = 118.26, *p* < 0.0001). Mantids captured and consumed butterflies with DS eyespots faster than butterflies with WS eyespots regardless of form (DS form with DS eyespots 2.6 ± 0.5 days, WS form with DS eyespots 2.8 ± 0.5 days, DS form with WS eyespots 7.8 ± 0.4 days, WS form with WS eyespots 11.1 ± 0.6 days, *F*_1,44_ = 4.73, *p* = 0.0351; figure 3*a*). The WS form laid more eggs than the DS form controlling for eyespot treatment, but it was marginally significant (DS form 13.3 ± 1.4 eggs, WS form 18.2 ± 2.1 eggs, *F*_1,44_ = 3.88, *p* = 0.0666). Butterflies with WS eyespots laid more eggs regardless of form (DS eyespots 2.6 ± 0.4 eggs, WS eyespots 29.0 ± 0.8 eggs, *F*_1,44_ = 10.72, *p* < 0.0001). The interaction effect between form and eyespots had a greater impact on WS form than DS form (DS form with DS eyespots 3.5 ± 0.7 eggs, WS form with DS eyespots 1.6 ± 0.2 eggs, DS form with WS eyespots 23.2 ± 0.5 eggs, WS form with WS eyespots 34.9 ± 0.4 eggs, *F*_1,44_ = 6.84, *p* = 0.0122; figure 3*b*). The WS form experienced greater, albeit marginally significant, ventral hindwing damage (DS form 66.91 ± 2.71%, WS form 73.50 ± 4.34%, *F*_1,44_ = 1.835, *p* < 0.0733). WS eyespots regardless of form had much greater ventral hindwing damage (DS eyespots 17.92 ± 2.64%, WS eyespots 82.29 ± 2.64%, *F*_1,44_ = 24.647, *p* < 0.0001). The interaction between form and eyespot was not significant for per cent hindwing damage (*F*_1,44_ = 1.19, *p* = 0.2379; figure 3*c*).

### (d) Eyespots are adaptive by deflecting invertebrate predator attack

We have demonstrated larger, brighter eyespots of the WS form of *B. anynana* are easier for an invertebrate predator to detect (figure 1*b*). Once detected, ventral hindwing eyespots direct predator attack to the wing margins, away from vital butterfly body parts, independently of butterfly form (figures 1*d*,*e*, 2*c* and 3*c*). Mantids attack and damage the largest eyespot on the ventral hindwing most frequently (Cu1) (figure 1*e*). Eyespots increase butterfly longevity and reproductive success when mantid predators are present in the local environment (figures 2*a*,*b* and 3*a*,*b*).

Past research indicated the large eyespots of WS *B. anynana* were not effective at deflecting vertebrate predator attack [7,8,10]. WS *B. anynana* were easier for vertebrate predators to detect; however, predators did not re-direct their attacks to the hindwing margin and WS *B. anynana* did not seem to accrue any fitness benefits for having large eyespots [7,8,10]. In these experiments, only approximately 4% of the numerous encounters between *B. anynana* and various vertebrate predators resulted in WS escape [7,8,10]. The discrepancy between vertebrate and invertebrate predator response to eyespots may be related to differences in the visual and nervous systems of the various experimental predators, or to the varying distances and angles from which distinct predators initiate their attack. Whatever the causes, mantids are misled by eyespot patterns more readily than vertebrate predators and alter their attack behaviour in ways that benefit survival and reproduction in WS *B. anynana*.

Across butterflies, eyespots have limited deflective effect on vertebrate predation under very specific light conditions. Low light conditions with accentuated UV promoted more avian attack to the wing margins of *Lopinga achine* [9]; however, these same butterflies suffered increased attacks under normal daylight conditions. Low light conditions are thought to increase the UV reflection of the white eyespot centre making the marginal eyespots more conspicuous to avian predators [9]. These findings are not transferable to mantids since mantids have monochromatic vision and are unable to see in the UV range [21], but the low light conditions may be relevant for other invertebrate predators, such as wasps, robber flies and spiders, which perceive signals in the UV range. More research is needed to understand the importance of predator identity, light conditions and their interaction on butterfly reproduction and survival.

Field surveys and mark recapture studies suggest butterfly and moth eyespot patterns deflect predator attack and are under selection in the wild. Individuals with more or larger eyespots have a higher recapture frequency and more damaged eyespots when recovered [22–24]. Also, the tensile strength of the wing regions where hindwing eyespots are found is often weaker than other areas of the wing and more easily torn [25,26]. These indirect observations have been attributed to selection by avian predators [22–26], but they are also consistent with pressure by invertebrate predators such as mantids. Mantids make similar damage patterns to beak marks and their attack has been noted for its speed and strength [13,27] (electronic supplementary material, video). Eyespot patterns on lepidopteran wings are probably under selection by a diverse community of both vertebrate and invertebrate predators.

Eyespots are found in a variety of other animals suggesting this colour pattern is of general adaptive importance. Investigations in marine and aquatic environments indicate eyespot patterns on fishes are used for predator intimidation or sexual attraction [21,28,29]. There remains at least some evidence eyespot may serve a deflective function. Fully reproductive butterfly fish are found in wild populations with up to 10% of their posterior body missing where eyespots are normally found [30]. Experiments using model prey found eyespots can attract attack by fish predators [31]. The deflection function may be working in conjunction with intimidation and sexual attraction in these systems. We hope our approach provides a useful experimental framework of combining ritualized predator–prey interactions with more general measurements of fitness for elucidating the various functions of eyespots across animals.

## 4. Conclusion

We leveraged a naturally occuring polyphenism in eyespot size to demonstrate larger, brighter eyespots deflected predator attack away from the more vulnerable body thereby increasing prey fitness. These marginal hindwing eyespots of WS *B. anynana* butterflies were more conspicuous to mantid predators; yet, these eyespots conferred greater survival and reproductive success compared to individuals with reduced, duller eyespots. Our results shed new light on the evolution of seasonal polyphenisms in *B. anynana* and other butterflies. Since ventral hindwing eyespots are beneficial to butterflies in the presence of invertebrate predators, these increases in reproduction and survival may offset the detection costs incurred against vertebrate predators [8]. Differences in phenology and longevity between invertebrate and vertebrate predators may explain the evolution of seasonal polyphenisms in eyespot size and colour.

Seasonal variation in eyespot size and other protective coloration has been primarily attributed to seasonal variation in avian predator intensity (reviewed in [1,2]). In *B. anynana*, the WS form is thought to have evolved as a result of relaxed avian predation during the rainy season coupled with increased avian predation in the dry season to produce the DS form [23]. Our results indicate increased invertebrate predation pressure may select for large, bright eyespots during the wet season while vertebrate predation pressure may select for small, dull eyespots in the dry season. This dynamic role of predator identity, their differences in vision, neurobiology and behaviour, in addition to predator abundance, may play an important role in the evolution of eyespot phenotypic plasticity.

## Supplementary Material

PrudicEtAl_PRSB_SupplFigure

## Supplementary Material

PrudicEtAl_PRSB_Data 1

## Supplementary Material

PrudicEtAl_PRSB_Data 2

## Supplementary Material

PrudicEtAl_PRSB_Data 3

## Data accessibility

Data available from the Dryad Digital Repository: http://doi.org/10.5061/dryad.kr85.
