# Supplementary figures and images for: Eyespots deflect predator attack increasing fitness and promoting the evolution of phenotypic plasticity
Source: Proc Biol Sci. 2015 Jan 7;282(1798):20141531. doi: 10.1098/rspb.2014.1531 (PMC4262162; doi:10.1098/rspb.2014.1531)

**(a)**

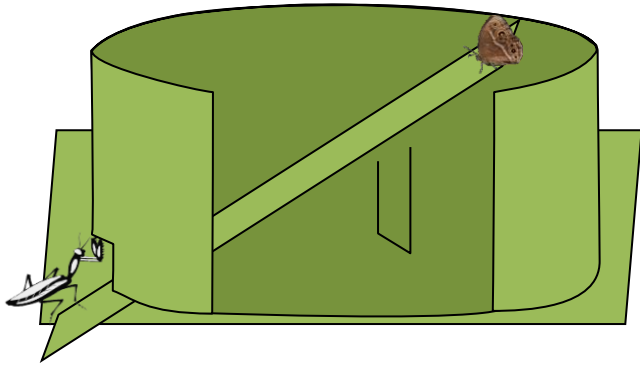

**(b)**

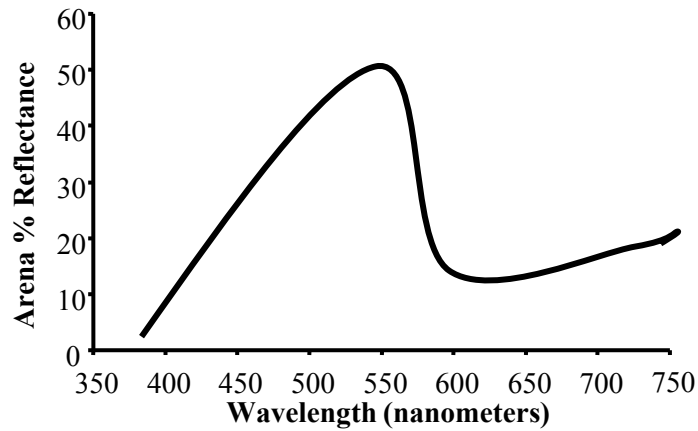

**(c)**

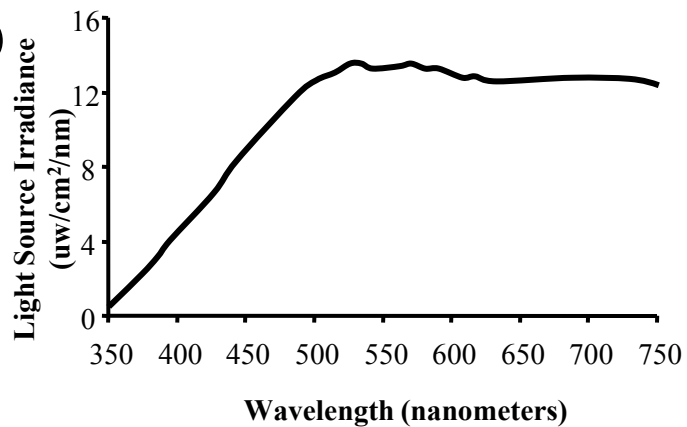

**(d)**

**Wet Season Form**

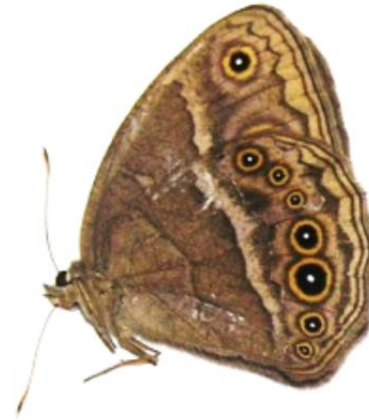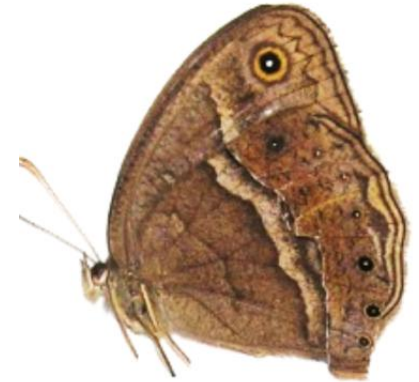

**Dry Season Form**

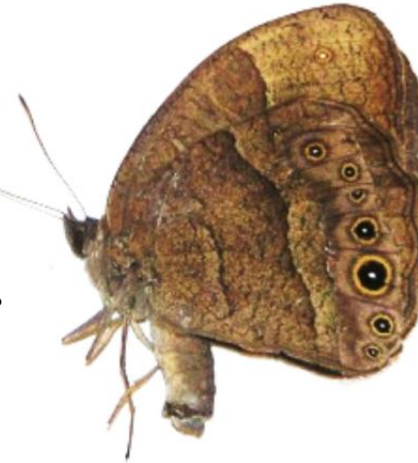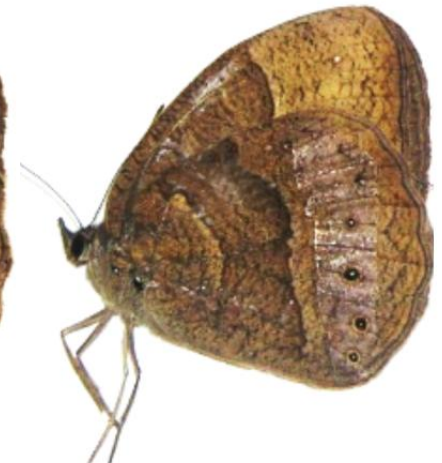

**Wet Season Eyespot**

**Dry Season Eyespot**

Suppl figure 1

Supplement: PrudicEtAl_PRSB_SupplFigure [file rspb20141531supp1.pdf]
